# The Utilization of Artificial Neural Network Equalizer in Optical Camera Communications †

**DOI:** 10.3390/s21082826

**Published:** 2021-04-16

**Authors:** Othman Isam Younus, Navid Bani Hassan, Zabih Ghassemlooy, Stanislav Zvanovec, Luis Nero Alves, Hoa Le-Minh

**Affiliations:** 1 Optical Communications Research Group, Faculty of Engineering and Environment, Northumbria University, Newcastle upon Tyne NE1 8ST, UK; z.ghassemlooy@northumbria.ac.uk (Z.G.); hoa.le-minh@northumbria.ac.uk (H.L.-M.); 2 Institute of Photonics, University of Strathclyde, Glasgow G1 1XQ, UK; navid.bani-hassan@strath.ac.uk; 3 Department of Electromagnetic Field, Faculty of Electrical Engineering, Czech Technical University in Prague, 16627 Prague, Czech Republic; xzvanove@fel.cvut.cz; 4 Instituto de Telecomunicações and Departamento de Electrónica, Telecomunicações e Informática, Universidade de Aveiro, 3810-193 Aveiro, Portugal; nero@ua.pt

**Keywords:** CP 4-PAM, optical camera communications, ANN equalizer

## Abstract

In this paper, we propose and validate an artificial neural network-based equalizer for the constant power 4-level pulse amplitude modulation in an optical camera communications system. We introduce new terminology to measure the quality of the communications link in terms of the number of row pixels per symbol $N_{\mathrm{pps}}$, which allows a fair comparison considering the progress made in the development of the current image sensors in terms of the frame rates and the resolutions of each frame. Using the proposed equalizer, we experimentally demonstrate a non-flickering system using a single light-emitting diode (LED) with $N_{\mathrm{pps}}$ of 20 and 30 pixels/symbol for the unequalized and equalized systems, respectively. Potential transmission rates of up to 18.6 and 24.4 kbps are achieved with and without the equalization, respectively. The quality of the received signal is assessed using the eye-diagram opening and its linearity and the bit error rate performance. An acceptable bit error rate (below the forward error correction limit) and an improvement of ~66% in the eye linearity are achieved using a single LED and a typical commercial camera with equalization.

## 1. Introduction

Optical camera communication (OCC) systems, which are part of the optical wireless communications (OWC), leverage the use of off-the-shelf conventional, complementary metal-oxide-semiconductor (CMOS) image sensors (ISs) and standard light-emitting diodes (LEDs) as the receiver (Rx) and the transmitter (Tx), respectively. 

The camera-based Rxs can capture intensity-modulated light signals from a range of LED light sources (i.e., traffic lights, advertising boards, signage, display screens, vehicle head, and taillights, streetlights, etc.). The OCC technology together with the visible and infrared light transmission could be used in different low data rate *R_b_* applications, such as the Internet of Things (IoT) (e.g., as part of the fifth-generation wireless and beyond), motion capturing [1], intelligent transportation systems [2], indoor localization, security, virtual reality, and advertising [3]. OOC comprises a plurality of pixels (i.e., photodetectors (PDs)), where the signal strength of each pixel depends on the intensity of incident light [4]. Each pixel can detect signals at different wavelengths over the visible range, e.g., red, green, and blue (RGB), hence offering parallel detection capabilities and an adaptive field of view (FoV) feature. In addition, the transmitted information from many light sources, different directions, and locations via the line-of-sight (LOS) [5,6], non-LOS, and/or a combination of both paths [7] can be captured using a single-pixel or a pixel-array IS-based Rx. Thus resulting in a higher signal-to-noise ratio, improved mobility, and flexibility over a linkspan up to hundreds of meters [8]. 

On the contrary, the IS requires a higher sampling duration and lower number of quantization levels compared with the PDs due to the light integration time (known as the exposure time $T_{\exp}$), and the built-in analog to digital converter circuit [9]. The sequential-readout nature of CMOS IS-based Rx allows each pixel-row to capture the incident light at a different time, thus resulting in the so-called rolling shutter (RS) effect [9]. Note that, the performance of VLC with IS-based Rx is limited mainly by the camera capabilities, i.e., the frame rate *R_f_*, $T_{\exp}$, and FoV. As a result, in OCC, the transmission bandwidth is rather low and limited to a few tens of kHz compared to the PD-based VLC systems. Although, low data should not be seen as a problem considering that there are many applications where low *R_b_* is not critical at all (i.e., IoT, etc.). However, in OCC, lower *R_b_* may result in the flickering effect at the Tx [10,11]. In IEEE 802.15.7m standard [12], different schemes have been proposed for OCC to mitigate flickering and to increase *R_b_* [13]. For example, in [14], an optical orthogonal frequency division multiplexing VLC with a special IS-based Rx with a built-in PD-array was used to achieve a very high *R_b_* of 55 Mbps. However, the fabrication process of the IS was too complex and, therefore, not commercially available. In [15,16], under-sampled frequency and phase shift on-off keying (OOK) modulation schemes were proposed to mitigate flickering in OCC with low *R_b_*. In [17], Manchester coding was proposed to alleviate flickering in the RS mode, where it was shown that link performance in terms of *R_b_* deteriorated with the transmission range [8,17]. 

Moreover, an OCC link with the under-sampled pulse amplitude modulation (PAM) with subcarriers was experimentally demonstrated with the increase *R_b_* to 250 bps [18,19]. In addition, a multilevel-intensity modulation scheme for RS-based OCC with the frame rate *R_f_* of 30 fps was proposed in [20] with *R_b_* of 10 kbps over a link range of up to 2 m. Furthermore, a parallel transmission VLC system with color-shift-keying (i.e., different colors RGB-LEDs) was reported in [21] with an overall *R_b_* of 5.2 kbps. In [22], the concept of parallel transmission was demonstrated over a range of up to 60 m and with *R_b_* of 150 bps. Whereas a 16 × 16 array μLED and a high-speed camera with *R_f_* of 960 fps and using *R_b_* of 122.88 kb/s was reported in [23]. 

In OCC systems, equalization methods can also be deployed to compensate for spatial and temporal induced dispersion. In [24], an OOK VLC (a single LED) and camera-based Rx with a dual equalization scheme to compensate for both spatial and temporal dispersion were reported with increased *R_b_* up to 14.37 kb/s. The artificial neural network (ANN) architecture has also been proposed for post-equalization to combat non-linear impairments in OWC [25,26]. The use of an ANN-based equalizer is one of the remarkable solutions adapted in PD-based OWCs, wherein the ANN act as the universal classifiers [27]. In [25], a 170 Mb/s OOK VLC link using an LED with a modulation bandwidth of 4.5 MHz and the ANN-based equalizer at the Tx was reported, where the superiority of ANN equalizers in mitigating intersymbol interference (ISI) was demonstrated compared with other equalization techniques. Note, in OCC with the ANN-based equalizer, the network needs to be trained once for a range of *T*_exp_ with the data being stored in a look-up table within the camera. 

In [28], the variable transparent amplitude shape code (VTASC) scheme was experimentally evaluated for device-to-device (D2D) (i.e., smartphones) communications in the form of high-density modulation (HDM) with the ANN assisted demodulator. *R_b_* of 2.66 Mbps over a 20 cm long transmission link was achieved. Note, the concept of D2D is one form of the multiple-input multiple-output system, where every pixel is transmitted and detected. Similarly, to allow transmission and reception of information under bad weather conditions, a convolution neural network (CNN)-based OCC was proposed in [29]. The CNN was used for classification and recognition of LED patterns and to decode the transmitted data streams even under an unclear state, where LED patterns are not visible to the camera due to blocking of the transmission path and/or weather conditions. In [30], an OCC link with an ANN-based decoder was reported to mitigate the gap-time effect between two adjacent frames, where OOK was transmitted using an RGB-LED with *R_b_* of 47 kb/s. In [4], an OCC link using a single LED source and Manchester line code with the non-return to zero formats was reported with *R_b_* of 14 kb/s. 

In this work, the aim is to establish a flickering-free OCC system with improved *R_b_* using a single LED and an ANNs-based equalizer. The key contributions extended from our previous work [31] are:

- Comprehensive and systematical investigation of the applicability of CP-PAM for the LED- and camera-based VLC.
- Development of a practical CP-PAM OCC prototype with a single Luxeon Rebel white LED (SR-01-WC310) and an IS (Thorlabs DCC1645C) as the Tx and the Rx, respectively.
- Development of an efficient signal extraction algorithm for the RS-based OCC system.
- Implementation of an ANN-based equalizer at the Rx to enhance the system performance.
- Development of an experimental test-bed for the proposed system and evaluating it in terms of the Tx’s frequency, eye diagrams, and the bit error rate (BER) with and without the ANNs-based equalizer.
- Proposing a new measurement metric for assessing the quality of the communications link in terms of the number of row pixels/symbol.

The remainder of the paper is organized as follows. Section 2 introduces the proposed CP PAM scheme, whereas Section 3 outlines the ANN equalizer model for IS-based OCC. The experimental setup is described in Section 4. Results and discussion are presented in Section 5. Finally, conclusions are given in Section 6.

## 2. Constant Power-PAM in RS-Based OCC System

The OCC system is mainly composed of a light source-based Tx with normalized length (diameter) represented in L, and a camera-based Rx, which is modeled using a single convex lens with a focal length *f*. The transmission speed in the RS-based OCC system is defined by the amount of the information that can be captured by an image at the distance d, which depends on the acquired number of samples (i.e., pixel rows) and is given by [32]:

(1) $$N_{\mathrm{row}}=2\;f\times tan\left(\frac{\mathrm{FoV}}{2}\right)=2\;f\times \frac{L}{2d}\;,$$

where FoV is the angular field of view.

Note that the acquired $N_{\mathrm{row}}$ is incorporated with the sampling frequency of the IS, known as the rolling rate of IS, $F_{s}$ (i.e., the frequency at which the row pixels are sampled at the image plane). 

Therefore, the maximum frequency of the transmitted signal is limited $\frac{F_{s}}{2}$ according to Nyquist’s theorem. The $F_{s}$ value depends on the pixel clock and *T*_exp_ (i.e., the time that every sample (pixel) of the IS is exposed to the light). Note, *T*_exp_ acts as a moving-average filter [10,33] with the frequency resolution given by:

(2) $$\Delta f=\frac{1\;}{T_{\exp}}=\frac{\;F_{s}}{N_{\mathrm{row}}\left(d\right)}\;,$$

$F_{s}$ is defined in terms of the bandwidth of the transmitted signal $f_{\mathrm{Tx}}$ and the number of received pixels per symbol, $N_{\mathrm{pps}}$, which is given by:

(3) $$F_{s}=N_{\mathrm{pps}\;}.\;\;f_{\mathrm{Tx}}\;,$$

Note, (*i*) $N_{\mathrm{pps}}$ varies with the payload *P*_bit_; and (*ii*) the maximum transmission distance is proportional to both Δf and the size (diameter) of the light source. Higher *T*_exp_ results in increased signal intensity levels, and, therefore, higher signal-to-noise-ratio (SNR) at the cost of reduced Rx bandwidth. With reference to Equations (1)–(3), a communications link can be established at low *R_f_* but with flickering, which is due to the variation in the mean value of light intensity during a time period larger than the optical bandwidth of the human eye. This may occur provided there are many consecutive symbols with the same logical state. 

**Figure 1:**
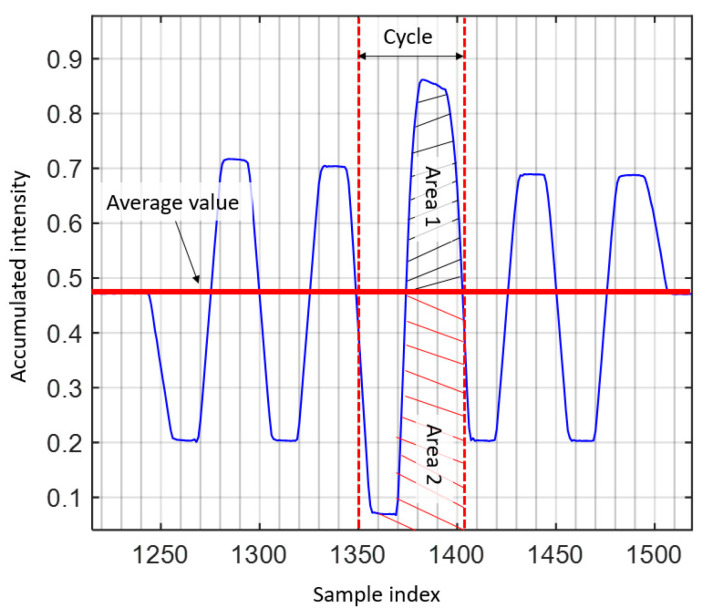
Defining Flicker Index [34,35].

The flicker index is a relative measure of the cyclic variation in the output of various sources at given frequencies [34,35]. It considers the waveform of the light output and its amplitude, which can be determined by dividing the area above the line of average light output by the total area under the light output curve for a single curve, see Figure 1, and is given by:

(4) $$\mathrm{Flicker}\;\mathrm{index}=\frac{\mathrm{area}\;1\;}{\mathrm{area}\;1+\mathrm{area}\;2}\;,\;$$

The flicker index has a range of 0 to 1.0, with 0 representing the steady light output level. Area 2 may be close to zero provided the light output varies as periodic spikes, thus leading to a flickering index close to 1. Higher values indicate an increased possibility of noticeable flickering.

**Figure 2:**
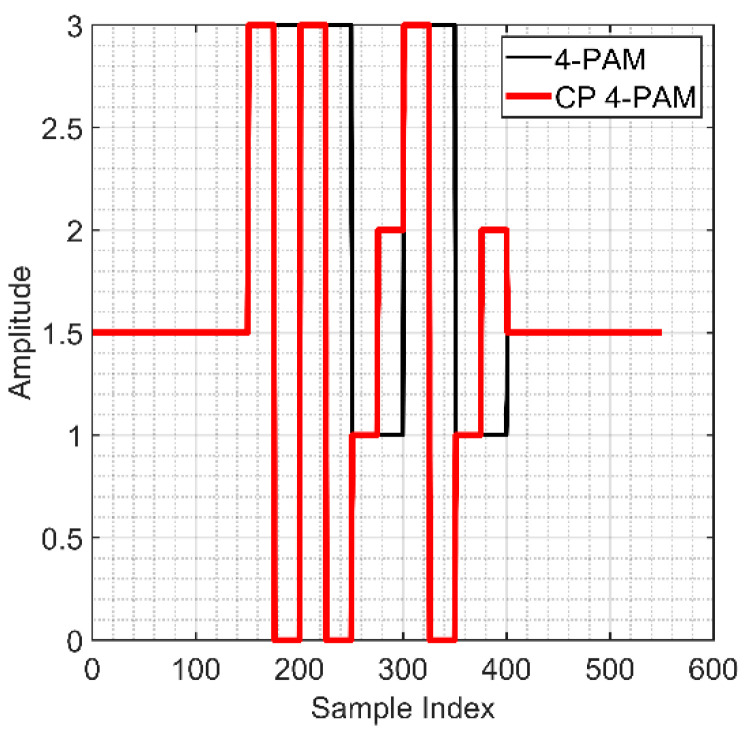
An example of a generated packet signal with $f_{\mathrm{Tx}}$ of 220 Hz.

To mitigate flickering, CP-PAM can be adopted to equalize the mean intensity value of all symbols, i.e., $I_{\mathrm{ave}}$ [18]. In CP-PAM, each PAM symbol is temporally divided into two equal chips, (*i*) the 1st chip for the intensity of the PAM symbol $I_{S}$; and (*ii*) the 2nd chip for the stabilization level, i.e., $2I_{\mathrm{ave}}-I_{S}$, see Figure 2. For example, a symbol with a level of “2” will be stabilized in the following chip with another symbol with a level of “1” to ensure performance equality, as clarified in Table 1.

It is also noted that considering the *R_b_* efficiency of CP 4-PAM is reduced by half due to the stabilization level (also used for error detection), the CP N-PAM offers a higher coding efficiency compared with Manchester coding [17]. 

## 3. ANN Equalizer

In RS-based OCC systems, the IS sampling process limits the available bandwidth and results in ISI at higher data rates, thus impacting the performance of the communications link. The ability to detect the slow rise-time symbol may be impacted by the existence of the transition between different illumination levels. Equalization is one option that is being adopted to mitigate the ISI. Note, the ISI is predicted by the training filter coefficients based on a training sequence. Alternatively, the ISI can be viewed as a classification problem, where class decision boundaries are created to classify symbols based on training [4]. Hence, determining the optimal threshold boundaries in a practical channel can be seen as a nonlinear process, and consequently, the ANN-based equalizer with the adaptive algorithm can be employed to mitigate ISI and, therefore, increase the data rate. Unlike other communication systems, OCC training of the ANN network is carried out only once for a specific exposure time with the data being stored with a look-up table [25,26].

An ANN is an interconnected network of processing elements (neurons). It comprises of two distinct stages: (i) The training phase, where the ANN estimates an input-output map between the received and training data to determine the weighted input from each neuron. The weighted values are updated in each training iteration until either the required performance is achieved, or the entire training set is used; and (ii) the operation phase, where the ANN is deployed without the knowledge of the dataset under test. The multilayer perceptron (MLP) is a popular ANN architecture, which has been demonstrated with high effectiveness in signal equalization [36]. It offers the ability to map any non-linear input-output sequence, provided there are sufficient neurons in the hidden layer(s), and the SNR is sufficiently high.

**Figure 3:**
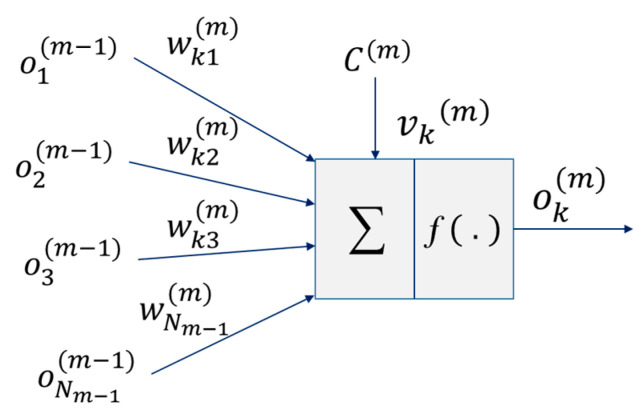
A structure of the *k*th neuron in the layer *m.*

The MLP structure consists of at least three layers; (*i*) a single input layer *x*; (*ii*) (M−1) hidden layers; and (*iii*) a single output layer *y*. The input layer (also called the observation vector) has the same structure as a conventional linear equalizer for sequential equalization, i.e., it is a tapped delay line ***o***^(*m*−1)^ = [$o_{1}^{\left(m-1\right)}$*,* $o_{2}^{\left(m-1\right)}$*, …,*$\;o_{N_{m-1}}^{\left(m-1\right)}$], where *N* is the number of neurons, and *m* is the layer number. This is illustrated in Figure 3, where weights $w_{kn}^{\left(m\right)}$ relate the *n*th input to the *k*th neuron. Each neuron can be biased with a value *C*^(*m*)^, which is in turn scaled by a threshold factor $v_{k}^{\left(m\right)}$. 

The output $o_{k}^{\left(m\right)}$ of the *k*th neuron is mapped via a non-linear activation function *f*(.) as given by [25]:

(5) $$o_{k}{}^{\left(m\right)}=f\left(\sum_{n=1}^{N_{m-1}}w_{kn}^{\left(m\right)}o_{n}^{\left(m-1\right)}+C^{\left(m\right)}v_{k}{}^{\left(m\right)}\right).$$

The output of each layer is usually connected to each of the neurons in the next layer, i.e., a fully connected mode, therefore, using the observation vector $o^{\left(m\right)}$ for the *m*th layer and the $N_{m}\times N_{m-1}$ connection matrix between layers m and m−1, the output is given in the vector form by:

(6) $$o^{\left(m\right)}=f\left(W^{\left(m\right)}o^{\left(m-1\right)}+C^{\left(m\right)}v^{\left(m\right)}\right),$$

where $W^{\left(m\right)}$ and $v^{\left(m\right)}$ are given by:

(7) $$W^{\left(m\right)}=\left[\begin{array}{l} w_{1}{}^{\left(m\right)}\\ w_{2}{}^{\left(m\right)}\\ .\\ .\\ .\\ w_{Nm}{}^{\left(m\right)} \end{array}\right],$$

(8) $$v^{\left(m\right)}={\left[v_{1}^{\left(m\right)}\;\;\;\;v_{2}^{\left(m\right)}\;\;\;\;\ldots \;\;\;\;v_{N_{m}}^{\left(m\right)}\right]}^{T}.$$

Considering the $N_{0}\times 1$ input vector, $N_{M}\times 1$ output vector, $o^{\left(0\right)}=x$ and $o^{\left(M\right)}=y$, the following observation vector $o^{\left(m\right)}$ is given by:

(9) $$\begin{array}{l} x={\left[x_{1}\;\;\;\;x_{2}\;\;\;\;\ldots \;\;\;\;x_{N_{o}}^{\left(m\right)}\right]}^{T}, \end{array}$$

(10) $$\begin{array}{l} y={\left[y_{1}\;\;\;\;y_{2}\;\;\;\;\ldots \;\;\;\;y_{N_{M}}\right]}^{T}. \end{array}$$

Therefore,

(11) $$\begin{array}{l} o^{\left(1\right)}=f\left[W^{\left(1\right)}x+C^{\left(1\right)}v^{\left(1\right)}\right), \end{array}$$

(12) $$\begin{array}{l} o^{\left(2\right)}=f\left[W^{\left(2\right)}o+C^{\left(2\right)}v^{\left(2\right)}\right), \end{array}$$

…

(13) $$\begin{array}{l} y=f\left[W^{\left(M\right)}o^{\left(M-1\right)}+C^{\left(M\right)}v^{\left(M\right)}\right). \end{array}$$

MLP will record its trained information in $w_{kn}{}^{\left(m\right)}$ and in the threshold factors $v_{n}{}^{\left(m\right)}$, since $C^{\left(m\right)}$ is given as a constant for all layers (i.e., set as $C^{\left(m\right)}=1,m=1,\;2,\ldots ,M$). Resilient back-propagation (RBP) is a supervised back-propagation (BP) training method, which updates the weights to converge more rapidly than the standard BP training technique [26]. Figure 3 depicts a single neuron for the case where the layers are interconnected with different weight coefficients. The RBP adjusts the MLP weights to reduce the error cost function $E_{n}$ as given by [25]:

(14) $$E_{k}=|\left|d_{k}-y_{k}\right|{|}^{2},$$

where $d_{k}$ and $y_{k}$ are the ideal and actual received symbols, respectively. It should be noted that, for the training sequence, *d* is known. Each iteration of the RBP algorithm has a dynamic step size, which varies based on the magnitude of the gradient descent of $E_{k}$.

## 4. Experimental Setup

**Figure 4:**
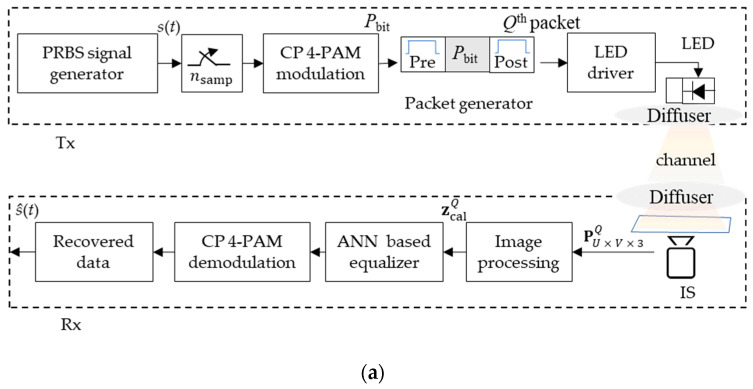

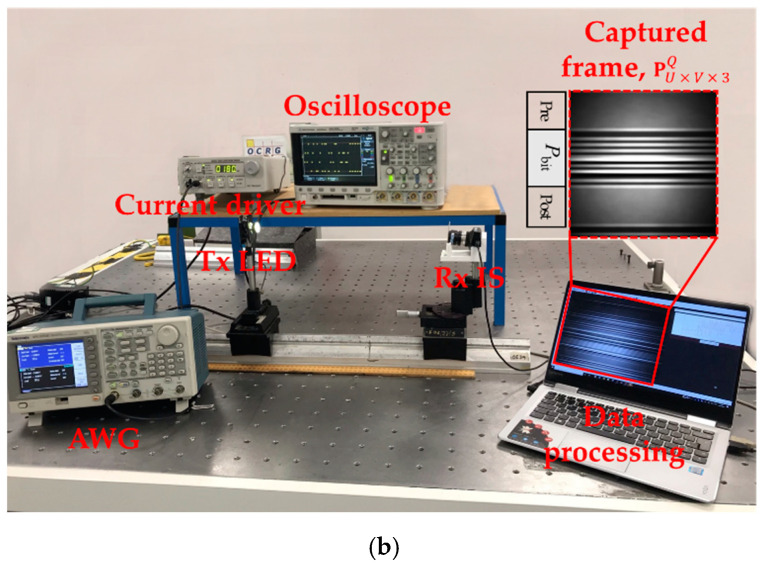
The CP 4-PAM OCC scheme: (**a**) System block diagram, and (**b**) photograph of the experimental setup.

The schematic block diagram of the proposed OCC system is shown in Figure 4a. A pseudorandom binary sequence (PRBS) with a length of $2{\;}^{16}$–1 bits was generated using MATLAB, which was then up-sampled with $n_{\mathrm{samp}}$ of 50 and modulated using CP-PAM. Based on the output labels provided for 4-PAM, see Table 1, mapping of the data to the corresponding symbols was carried out. The PRBS *s*(*t*) was divided into sub-sequences with effective symbols per packet with lengths of *P*_bit_-symbols, which depends on the transmitter bandwidth $f_{\mathrm{Tx}}$. Each subsequence was encoded with a pre- and post-amble to form a *Q*th Tx packet, where *Q*th represents the packet number and each packet consists of 3-symbol pre-amble [1.5 1.5 1.5], *P*_bit_-symbol payload, and 3-symbol post-amble [1.5 1.5 1.5]. 

The symbols in the overhead signal (i.e., pre-amble and post-amble) were chosen to ensure constant average optical power when compared with the payload. The signal was then sequentially uploaded onto an arbitrary wave generator (AWG, AFG3252C, 240 MHz bandwidth), see Figure 4b. The uploading process was done through the generation of *Q*th Tx packet at different $f_{\mathrm{Tx}}$, the output of which was used for intensity modulation of a Luxeon Rebel LED (SR-01-WC310) with a peak wavelength at 630 nm. Note, a linewidth of 118 nm was used for transmission of the modulated light over a short LoS free-space channel (i.e., 50 cm).

**Figure 5:**
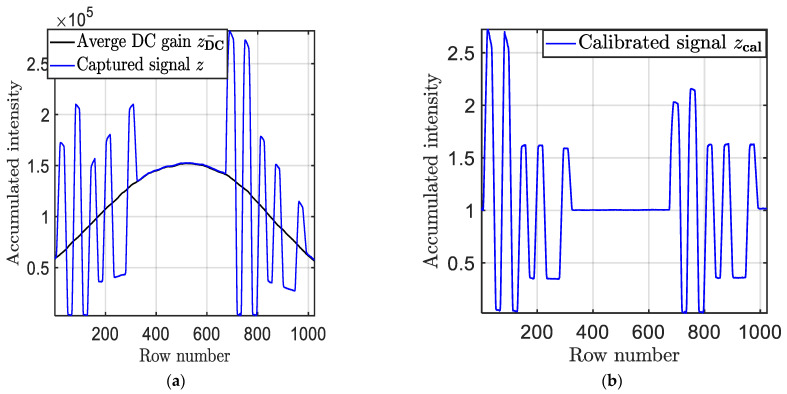
An example of the received *Q*th Tx packet signal at a *T*_exp_ of 2 ms: (**a**) without DC gain normalization, and (**b**) with normalization.

At the Rx, a diffuser was used to scatter the light over the capturing area of the IS (Thorlabs DCC1645C RS) with a standard *T*_exp_ of 2 ms was adopted in this study. The observed frames $P_{U\times V\times 3}^{Q}$ at the output of the camera were processed off-line in MATLAB using both Algorithms 1 and 2. In Algorithm 1, the data set $z_{i}{}^{Q}$ was retrieved by accumulating the intensities for all pixels in each row. The received signal was then normalized to remove the DC by capturing 20 frames with no signal, see Figure 5a,b.
**Algorithm 1** Signal Extraction Algorithm
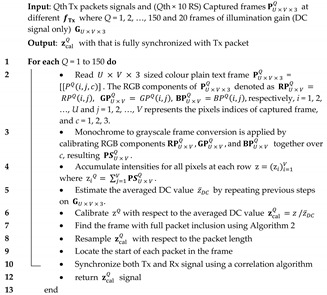


Next, to ensure that a full packet was captured by the IS, Algorithm 2 was applied to select the optimum frame (i.e., including both pre- and post-ambles) for each *Q*th Tx packet per $f_{\mathrm{Tx}}$, in which $10\times P_{U\times V\times 3}^{Q}$ are captured at *Q*th Tx packet to maintain the synchronization between both the Tx and the Rx.

A resampling process was then applied to resize the signal length based on the packet size observed in pixels. Next, a correlation algorithm was used to maintain the synchronization between the transmitted *Q*th Tx packet and received $z_{\mathrm{cal}\;}^{Q}$ signals, where a filtered version of $z_{\mathrm{cal}\;}^{Q}$ was simulated based on the encoded *Q*th packet using a moving average filter. Note, the window size of the filter was set to $n_{\mathrm{samp}}$ since it provided an optimal match compared with the observed signal. Next, $z_{\mathrm{cal}}$ in the vector form was applied to an MLP equalizer using an array of tapped-delay lines as previously described. The MLP used here included single input, hidden, and output layers. All the key system parameters are listed in Table 2.
**Algorithm 2** Find the frame with full packet inclusion (i.e., includes both pre- and post-ambles)
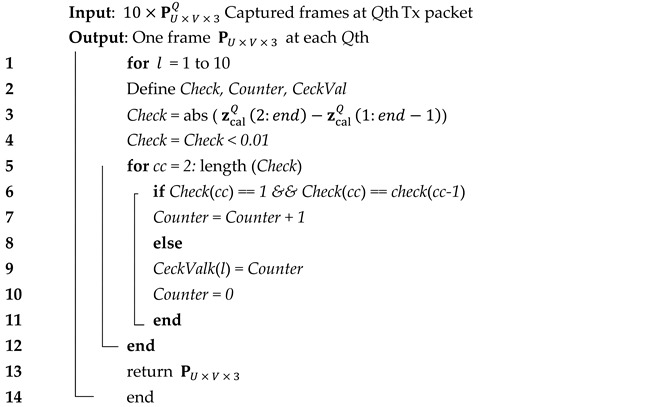


## 5. Results and Discussion

**Figure 6:**
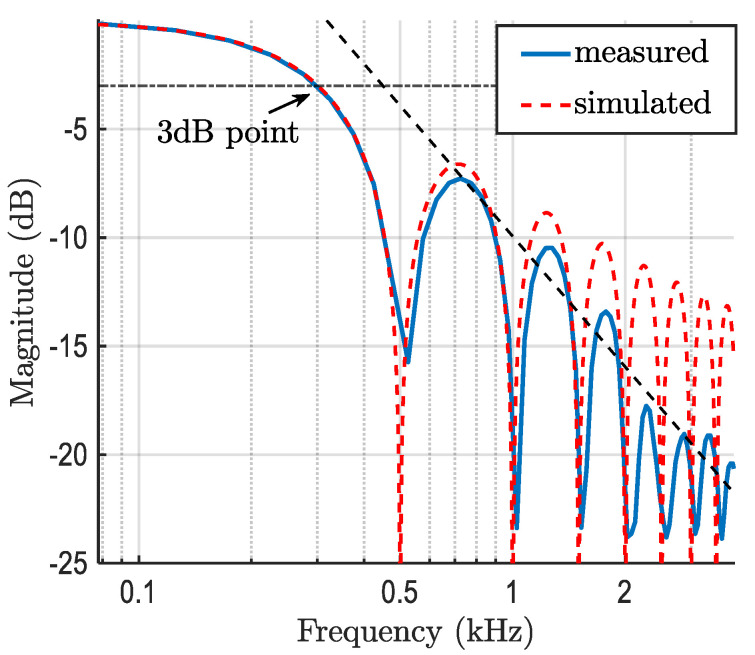
Measured and estimated bandwidth of the for IS with *T*_exp_ of 2 ms.

The experimental work was focused on deploying an MLP-based equalization to mitigate the ISI due to the limited modulation bandwidth of the CMOS IS-based Rx. The measured and simulated CIS for *T*_exp_ of 2 ms are highlighted in Figure 6, showing that the obtained IS bandwidth (i.e., a 3 dB point) was 250 Hz. It is also noted that the mismatch between the measured and simulated response was caused by aliasing due to the limited sampling frequency of the IS and utilization of image compression techniques [38]. The CP 4-PAM encoded signal was then generated at a different bandwidth $f_{\mathrm{Tx}}$ of up to 1520 Hz.

**Figure 7:**
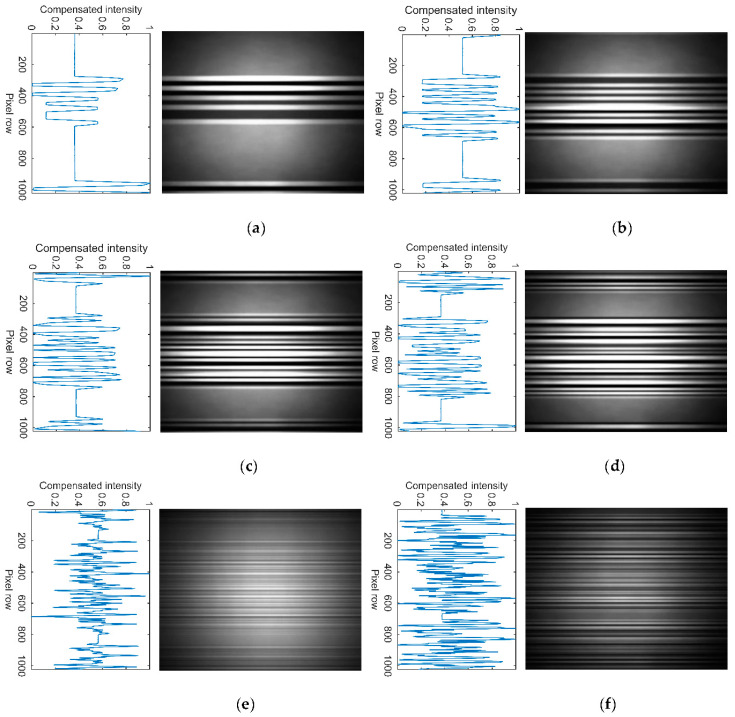
Examples of the frame acquisition based on CIS for CP 4-PAM and $f_{\mathrm{Tx}}$ of: (**a**) 220 Hz, (**b**) 320 Hz, (**c**) 420 Hz, (**d**) 520 Hz, (**e**) 1120 Hz, and (**f**) 1520 Hz.

**Figure 8:**
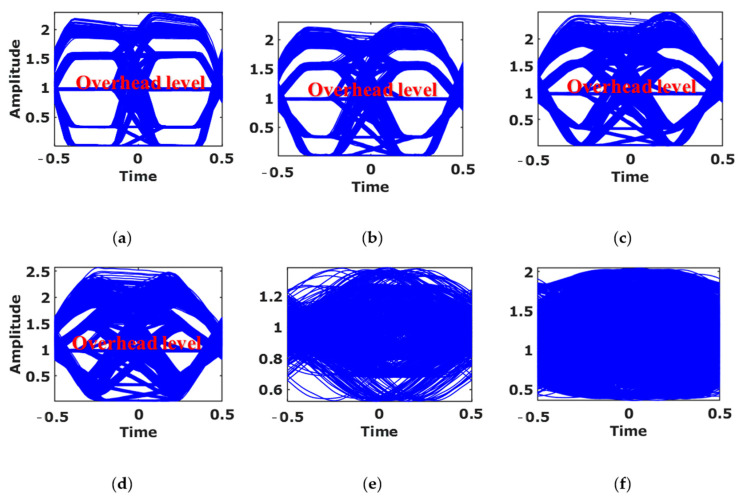
Examples of the captured eye diagrams of the CIS received signal for CP 4-PAM with $f_{\mathrm{Tx}}$ of: (**a**) 220 Hz, (**b**) 320 Hz, (**c**) 420 Hz, (**d**) 520 Hz, (**e**) 1120 Hz, and (**f**) 1520 Hz.

The captured frames at the Rx are processed with *P*_bit_ of up to 70 symbols per packet. Figure 7 illustrates examples of the captured frames and the processed signals for *P*_bit_ of 5, 10, 15, 20, 50, and 70, i.e., $f_{\mathrm{Tx}}$ 220, 320, 420, 520, 1120, and 1520 Hz, respectively. Note, the width of the received *Q*th packet and the recorded *F_s_* are 666 pixels and 13.31 kHz, respectively, based on the demodulated signal, see Figure 7. Increasing $f_{\mathrm{Tx}}$ decreases the number of received pixels for each CP 4-PAM symbol, thus, reducing the quality of data transmission. The number of pixels utilized for each CP 4-PAM symbol is indicated in Table 3. For the link with the ANN-based equalizer deployed at the Rx side, the quality of the received signal was measured using the eye diagrams and the BER performance. As illustrated in the eye diagrams, see Figure 8, the eye-openings indicate the impact of the ISI on the received signal. Note, (*i*) the threshold levels can be differentiated for *P*_bit_ of 5 and 20 symbols, see Figure 8a,b, respectively, but not for *P*_bit_ of 50 and 70 symbols as in see Figure 8c,d, respectively; (*ii*) the five levels are shown in the eye diagrams, where one of the levels represents the packet overhead designed to maintain the same average power for CP 4-PAM; and (*iii*) the overhead level is removed at the Rx side using Algorithm 1. 

**Figure 9:**
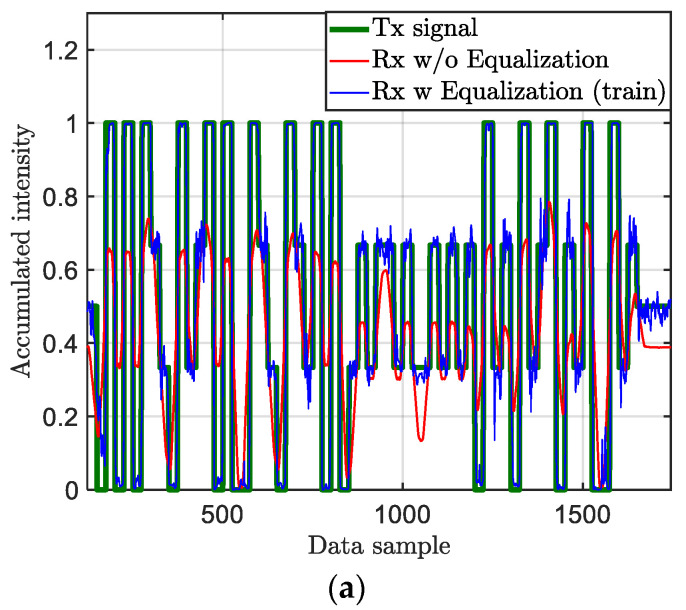

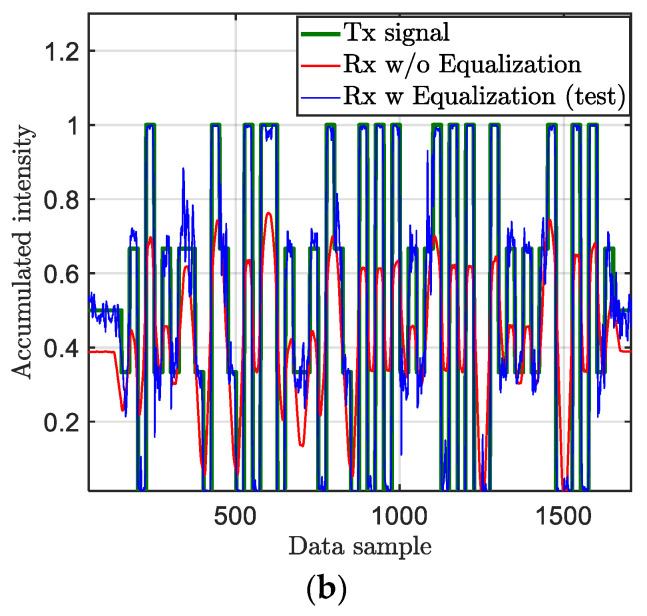
An example of the transmitted and received signal with and without equalization for CP 4-PAM and $N_{\mathrm{pps}}$ of 18.5 row pixels/symbol: (**a**) training sets, and (**b**) testing sets.

An example of transmitted and received signals with and without equalizer for $N_{\mathrm{pps}}$ of 8.7 pixels per symbol is illustrated in Figure 9. The equalized signal at the Rx side shows a significant improvement in reducing the impact of the ISI on the received signal with minimal signal distortions. 

The eye linearity of the received signals is measured based on the average amplitude levels is given by [39]:

(15) $$\mathrm{Eye}\;\mathrm{linearity}=\frac{\min\left(V_{\mathrm{up}},\;V_{\mathrm{mid}},\;V_{\mathrm{low}}\right)}{\max\left(V_{\mathrm{up}},\;V_{\mathrm{mid}},\;V_{\mathrm{low}}\right)}\;,$$

where $V_{\mathrm{up}},\;V_{\mathrm{mid}},\;\mathrm{and}\;V_{\mathrm{low}}$ are the average amplitude levels.

**Figure 10:**
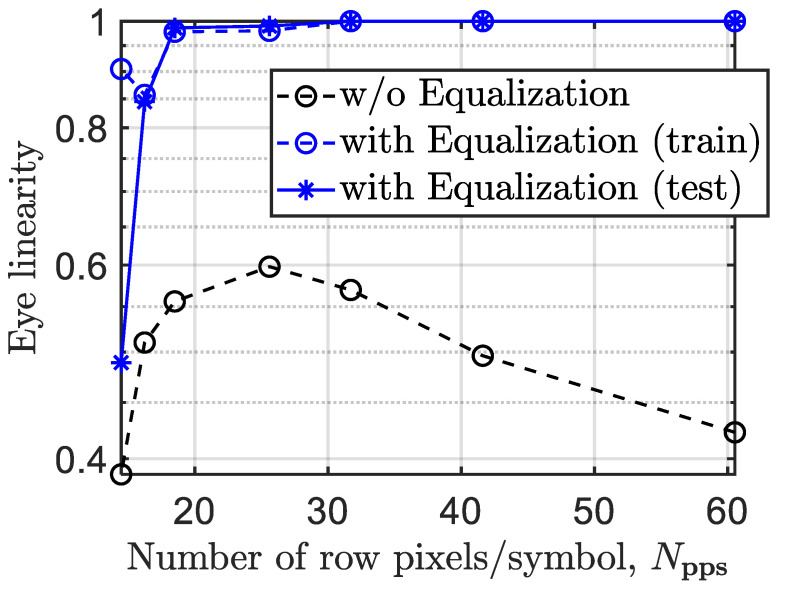
The eye linearity against $N_{\mathrm{pps}}$ for the proposed system with and without equalization and for *T*_exp_ of 2 ms.

Figure 10 shows the eye linearity of the received signals with respect to $N_{\mathrm{pps}}$ for the link with and without ANN equalizer and for *T*_exp_ of 2 ms. Note, we have used $N_{\mathrm{pps}}$, i.e., new terminology for a fair comparison considering the progress made in the development of ISs. As shown, for the link with no equalizer, the eye linearity increases with $N_{\mathrm{pps}}$ reaching a maximum level of 0.6 at $N_{\mathrm{pps}}$ of ~26, beyond which it drops linearly with rapidly $N_{\mathrm{pps}}$. However, with the ANN equalizer, the eye linearity is improved significantly for both the test and trained cases reaching the optimal linearization of almost 1 at $N_{\mathrm{pps}}$ of 18 and remaining constant beyond $N_{\mathrm{pps}}$ > 18 (i.e., being independent of $N_{\mathrm{pps}}$). Thus, the ANN equalizer show an improvement of ~66% in the eye linearity for $N_{\mathrm{pps}}$ > 18 pixels/symbol (i.e., $f_{\mathrm{Tx}}$ < 920 Hz) for both training and testing sets.

**Figure 11:**
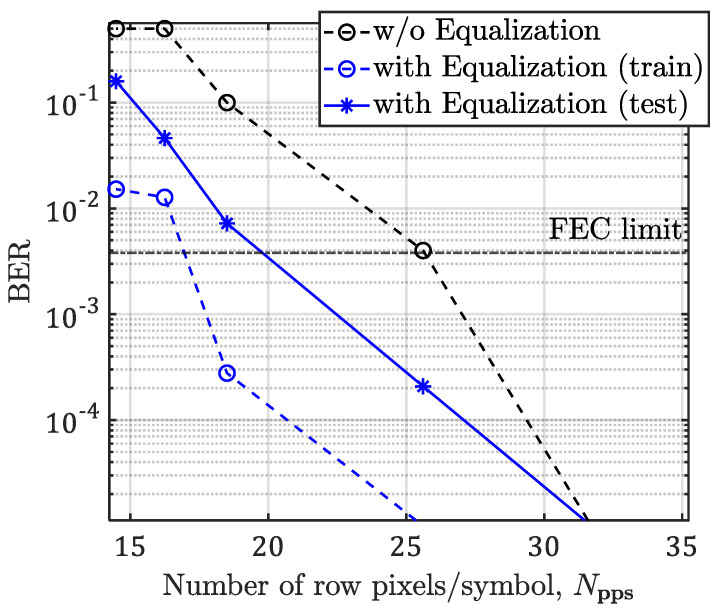
The BER measurements as a function of $N_{\mathrm{pps}}$ for the proposed system with and without equalization and for *T*_exp_ of 2 ms.

Next, we measured the BER as a function of $N_{\mathrm{pps}}$ for the link with and without ANN equalizer, as illustrated in Figure 11. In addition, shown is the forward error correction (FEC) BER limit line of 3.8 × 10^−3^. Note, at the FEC limit the $N_{\mathrm{pps}}$ value is reduced from 30 to 20 for pixels per symbol for the links without and with the ANN equalizer, respectively, compared with the test plot. Thus, the effective *R_b_* (i.e., no post- and pre-ambles) is estimated by:

(16) $$R_{b}=2\frac{V}{N_{\mathrm{pps}}}\cdot R_{f}\;,$$

where V represents the pixel row.

The effective $R_{b}$ at the FEC limit for the case with and without the ANN equalizer with a range of IS resolutions is indicated in Table 4. It demonstrates with the ANN equalizer *R_b_* of 24.4 and 12.2 kbps for *R_f_* of 60, and 30 fps, respectively, can be achieved compared with the case of no equalizer with *R_b_* of 18.6, and 9.3 kbps for *R_f_* of 60, and 30 fps, respectively. 

## 6. Conclusions

We proposed an ANN-based equalization technique for a CP 4-PAM based OCC system. An experimental setup was developed to demonstrate non-flickering communications using a single light-emitting diode with a transmission rate of *R_b_* of 24.4 kbps. The quality of received signals was measured based on the eye-diagram opening, eye linearity, and the BER. We demonstrated the ability to mitigate the intersymbol interference and hence to transmit a signal with an acceptable BER (below the FEC limit) for $N_{\mathrm{pps}}$ of 20, and 30 for unequalized and equalized systems, respectively. An improvement of ~66% in the eye linearity was achieved using a single LED, and a typical commercial camera with equalization technique was achieved. The limitation of the proposed system was assessed by the system complexity, including the associative memories needed for the look-up table training data as well as the IS resolution, gap-time and exposure time, and reading time.

## Figures and Tables

**Table 1:** Proposed CP 4-PAM levels.

Input Data	Conventional PAM Level	Constant Power 4-PAM	
		First Level $\left(I_{S}\right)$	Stabilization Level $\left(2I_{ave}-I_{S}\right)$
11	3	3	0
10	2	2	1
01	1	1	2
00	0	0	3

**Table 2:** System parameters.

Description		Value
**Tx**	LED type	Luxeon Rebel LED (SR-01-WC310)
	Tx signal bandwidth $\;\;f_{\mathrm{Tx}}\;$ (Hz)	220–1520 Hz
	Tx bias current	180 mA
**Camera Rx**	Camera model	Thorlabs DCC1645C-HQ
	Exposure time *T*_exp_	2 ms
	Maximum SNR of IS	44 dB [37]
	Lens type	Navitar 12 mm F/1.8 2/3” 10 MP
	Pixel clock	10 MHz
	Camera raw image resolution	1280 × 1024 pixels
	Captured symbols per frame	11–76 symbols
**Packet Generator**	Data format	CP-PAM
	Symbol per packet *P*_bit_	5–70 symbols
	Packet generator sample rate	11.125 kHz
	Number of samples $n_{\mathrm{samp}}$	10
**Channel**	Channel length	50 cm
**ANN Equalizer**	Activation function	Hyperbolic tangent sigmoid
	Number of neurons in input layer	200
	Number of neurons in output layer	1
	Number of neurons in hidden layer	200
	Number of hidden layers	2
	Percentage of the train to test	0.8
	Maximum epochs	1000
	learning rate parameter *η*	0.01
	Network training function	Resilient back-propagation

**Table 3:** Results with *R_f_* of 30 fps and CIS width of 1024 px.

Payload Symbol/Packet (*P*_bit_)	Total Number of Symbols/Packet	$\mathrm{Number}\;\mathrm{of}\;\mathrm{Row}\;\mathrm{Pixels}/\mathrm{Symbol}\;(N_{pps})$	$f_{Tx}\;\;(\mathrm{Hz})$
5	11	60.54	220
10	16	41.62	320
15	21	31.71	420
20	26	25.61	520
30	36	18.50	720
35	41	16.24	820
40	46	14.48	920
50	56	11.89	1120
70	76	8.76	1520

**Table 4:** Effective *R_b_* at different ISs resolutions at the FEC limits.

ISs Resolutions	$R_{b}\;(\mathrm{bps})\;\mathrm{at}\;N_{pps}=26$ (i.e., w/o Equalization)		$R_{b}\;(\mathrm{bps})\;\mathrm{at}\;N_{pps}=20\;$ (i.e., with Equalization)	
	*R_f_* = 30 fps	*R_f_* = 60 fps	*R_f_* = 30 fps	*R_f_* = 60 fps
1200 × 1800	3794	7588	5040	10,080
1500 × 2100	4486	8972	5940	11,880
1800 × 2400	5178	10,357	6840	13,680
2100 × 3000	6563	13,126	8640	17,280
2400 × 3000	6563	13,126	8640	17,280
3300 × 4200	9332	18,665	12,240	24,480

## Data Availability

The data presented in this study are available on request from the corresponding author.
